# Supplementary material for: Lesion site and therapy time predict responses to a therapy for anomia after stroke: a prognostic model development study
Source: Sci Rep. 2021 Sep 17;11:18572. doi: 10.1038/s41598-021-97916-x (PMC8448867; doi:10.1038/s41598-021-97916-x)
Supplement: Supplementary file 1 — Supplementary Information. [file 41598_2021_97916_MOESM1_ESM.docx]

Supplementary Table S1: Demographic and clinical data of the patients. There were 12 men, and 6 women; age at stroke mean = 50.4yrs; standard deviation = 11.5yrs.

| Patient ID | Lesion volume (cm^3^) | Months post-stroke | BNT | CAT | PALPA 9 | PALPA 8 | Hours of training | | |
| --- | --- | --- | --- | --- | --- | --- | --- | --- | --- |
| P1 | 171 | 78 | 47 | 15 | 20 | 6 | 40 | | |
| P2 | 44 | 17 | 12 | 15 | 21 | 6 | 31 | | |
| P3 | 294 | 78 | 14 | 11 | 10 | 0 | 77 | | |
| P4 | 234 | 65 | 28 | 14 | 24 | 8 | 116 | | |
| P5 | 144 | 57 | 34 | 15 | 17 | 2 | 50 | | |
| P6 | 109 | 61 | 52 | 15 | 24 | 6 | 63 | | |
| P7 | 82 | 72 | 34 | 14 | 24 | 10 | 59 | | |
| P8 | 95 | 34 | 35 | 15 | 24 | 8 | 70 | | |
| P9 | 341 | 47 | 42 | 14 | 24 | 9 | 85 | | |
| P10 | 75 | 8 | 23 | 13 | 23 | 8 | 89 | | |
| P11 | 139 | 264 | 51 | 15 | 24 | 9 | 81 | | |
| P12 | 314 | 52 | 16 | 15 | 22 | 6 | 77 | | |
| P13 | 150 | 40 | 1 | 14 | 18 | 2 | 61 | | |
| P14 | 104 | 121 | 27 | 13 | 22 | 7 | 120 | | |
| P15 | 114 | 18 | 42 | 14 | 21 | 3 | 43 | | |
| P16 | 155 | 33 | 18 | 15 | 20 | 3 | 108 | | |
| P17 | 161 | 53 | 9 | 9^a^ | 12 | 0 | 76 | | |
| P18 | 165 | 5 | 21 | 15 | 23 | 1 | 67 | | |
| Mean (SD) | **161 (84)** | **61 (58)** | **28 (15)** | **14 (2)** | **21 (4)** | **5 (3)** | **73 (25)** | |  |
|  | **Max score possible** | | | **60** | **15** | **24** | **10** |  | |
